# The Public Health

**Published:** 1918-10-19

**Authors:** 


### THE PUBLIC HEALTH.

### The Hospital Treatment of Pulmonary Tuberculosis.

MANY of the schemes for the treatment of tuberculosis which have been initiated since the coming into force of the National Insurance Act have not included anything like adequate provision for the hospital treatment of acute and advanced cases of pulmonary tuberculosis. The public mind was, and even yet continues to be, obsessed by the idea that in a brief residence of three months in a sanatorium is to be found the only method by which the successful treatment of pulmonary tuberculosis can be secured. And even medical men are not altogether immune from mistaken conceptions regarding the true *rôle* of the sanatorium in the scheme of treatment, and the type of case for which it was originally intended. The absence of adequate hospital accommodation has seriously impaired the true value of the sanatorium by compelling the admission of acute and active cases of tuberculosis to an institution which is only intended and organised for the treatment of early localised and quiescent cases of the disease. The hospital for pulmonary tuberculosis, provided the accommodation is adequate, serves a twofold important purpose. It provides immediate treatment for cases characterised by an acute onset, with a fair prospect of sufficient recovery to be subsequently removed to a sanatorium in a certain percentage of such cases. It further provides segregation for advanced cases, especially with laryngeal involvement, during the most infective phase of the disease, when efficient segregation cannot be provided at home. While the sanatorium plays its own important part in the treatment of tuberculosis, the problem of the prevention and treatment of this disease will never be successfully solved until greater attention is given to the important questions of housing and the provision of adequate hospital accommodation.

### War and Epidemic Disease.

THE possibility that the end of the war may be hastened by an outbreak of some acute epidemic disease in the armies of the enemy has not escaped the notice of medical men. While it is possible by means of inoculation to protect the health of the soldier against certain forms of epidemic disease, there are certain epidemic diseases, notably typhus fever, against which no such prophylactic treatment is at present known. The history of previous wars, and of the present war in Russia and Serbia, has shown to what extent typhus fever may be instrumental in claiming a high death-roll. To what extent the conditions which pave the way for a serious outbreak of epidemic disease in Germany and the German Army are likely to exist it is not possible accurately to determine, but all available evidence points to the fact that, as the duration of the war extends, the standard of feeding, physical condition, *morale*, and cleanliness of the German soldier is gradually depreciating, while the standard of efficiency of the Medical Service is not likely to be maintained at its former or pre-war level.

